# One-Year Outcomes Following Intravenous Ketamine Plus Digital Training Among Patients with Treatment-Resistant Depression: A Secondary Analysis of a Randomized Clinical Trial

**DOI:** 10.1001/jamanetworkopen.2023.12434

**Published:** 2023-05-08

**Authors:** Rebecca B. Price, Meredith L. Wallace, Sanjay J. Mathew, Robert H. Howland

**Affiliations:** 1 Department of Psychiatry, University of Pittsburgh School of Medicine, Pittsburgh, Pennsylvania; 2 Baylor College of Medicine and Michael E. Debakey VA Medical Center, Houston, Texas

## Abstract

This secondary analysis of a randomized clinical trial examines whether automated self-association training can prolong the antidepressant effect of a single infusion of ketamine beyond 1 month in patients with treatment-resistant depression.

## Introduction

In a recently published randomized clinical trial,^1^ we presented evidence that a novel digital intervention rooted in Pavlovian conditioning,^2^ automated self-association training (ASAT), can prolong the antidepressant effect of a single infusion of ketamine in patients with treatment-resistant depression. Brief computer exercises using positive words and images, designed to rehabilitate self-worth, created an effect on the primary clinician-rated depression outcome that persisted until the last in-person study visit at 1-month postinfusion. Given the lack of any waning effect at the final acute phase study visit, here, we utilized self-report data, collected during a 1-year naturalistic follow-up, to explore the full extent of the durability of this synergistic intervention.

## Methods

**Figure 1.**
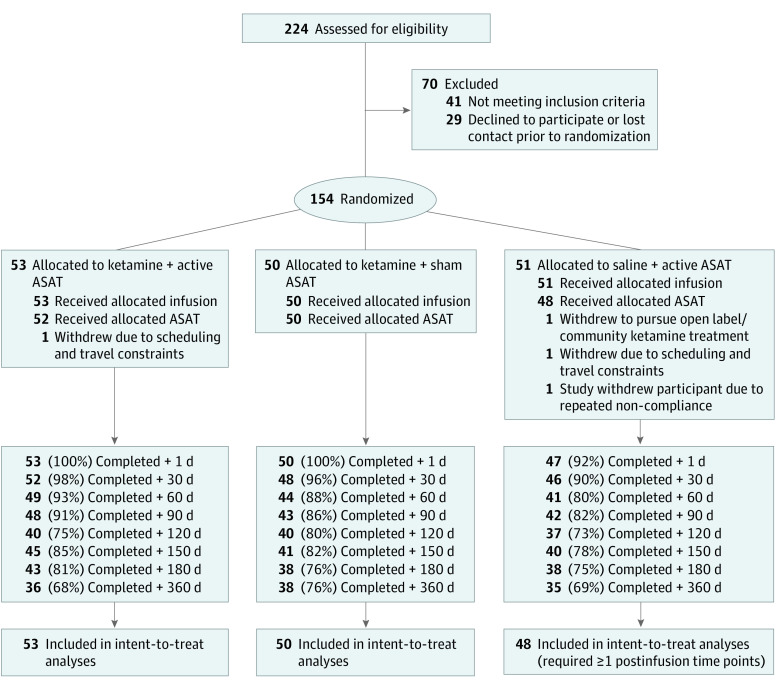
Study Flow Diagram Patients are labeled as having received their allocated infusion if they initiated the infusion. For 1 single patient, who was allocated to the ketamine plus sham automated self-association training (ASAT) group, the infusion was discontinued after administration of approximately two-thirds of the intended dose, at the patient’s request. Patients are labeled as having received their allocated ASAT condition (active or sham) if they completed at least 75% of the intended 8 sessions. All but 2 of the patients labeled as having received their ASAT allocation completed the entire set of 8 sessions, per protocol. During the follow-up period, all missing data are attributable to patients who were lost to follow-up or patients who withdrew (as described under Allocation). Missing data values were automatically estimated within the presented intent-to-treat hierarchical linear models. Missingness of data did not differ as a function of treatment allocation at any time point according to χ^2^ tests.

This secondary analysis of a randomized clinical trial followed the CONSORT reporting guideline. The study protocol is in Supplement 1. In the parent study, 154 adult patients with treatment-resistant depression, on stable psychiatric treatments, were randomized to 1 of 3 treatment conditions^1^: a single infusion of ketamine (0.5 mg/kg) followed by 4 days (30-40 min/d) of ASAT, a digital therapeutic we designed to leverage a hypothesized window of plasticity following ketamine to target implicit thought patterns related to oneself; ketamine followed by sham ASAT (identical computer exercises lacking therapeutic and self-relevant content); or saline infusion followed by active ASAT (Figure 1; eMethods in Supplement 2). Throughout a 1-year naturalistic follow-up period planned a priori (ClinicalTrials.gov identifier: NCT03237286), patients completed the Quick Inventory for Depressive Symptoms (QIDS-SR^3^), designated as the primary outcome during the follow-up phase. Surveys were solicited at the following time points after the infusion date: 30, 60, 90, 120, 150, 180, and 360 days.

**Figure 2.**
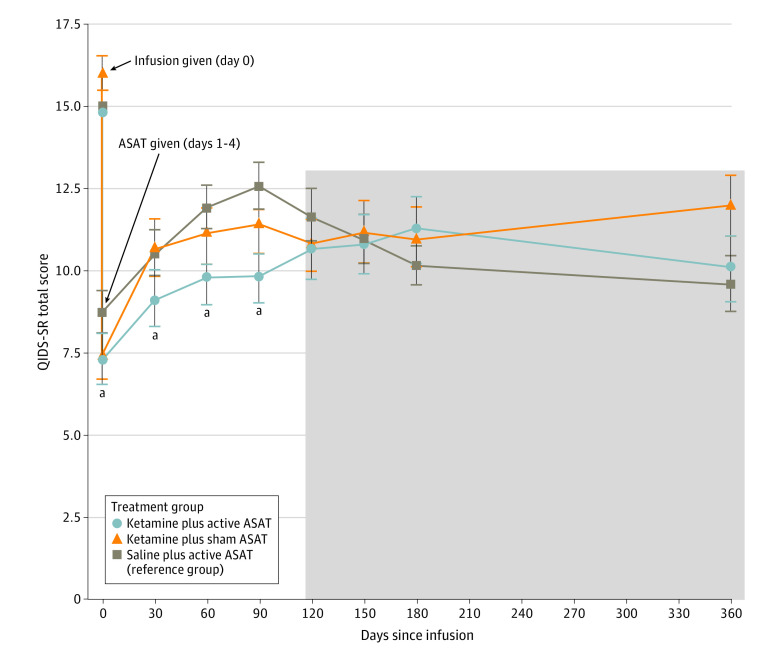
Depression Severity Scores as a Function of Days Since Infusion and Treatment Allocation Total Quick Inventory of Depressive Symptoms: Self-Report (QIDS-SR) scale scores are displayed for each treatment group: (1) ketamine plus active automated self-association training (ASAT) (n = 53); (2) ketamine plus sham ASAT (n = 50); and (3) saline plus active ASAT (n = 51; reference group for analyses). Line plots represent mean values and error bars represent standard error of the mean within each time point and treatment group. Gray shading indicates late timeframe where no significant pairwise group differences were observed at any time point, between any 2 groups. Preinfusion baseline and 1-day postinfusion (immediate postketamine or postsaline, prior to the onset of active/sham ASAT) are depicted here for comprehensive visualization, but the current follow-up period statistical analyses focused on the period from 30 days postinfusion and forward (as described in the text). ^a^ Indicates time point with significant decrease in ketamine plus active ASAT group relative to saline plus active ASAT group per unpaired *t* tests.

Time point and group (using saline plus ASAT as the reference group) were included as categorical factors in an intent-to-treat, hierarchical linear model (which automatically estimates missing values) estimating QIDS-SR total scores, covarying preinfusion baseline QIDS-SR scores. To dissect group × time interactions, given lack of a priori knowledge regarding potential durability of this novel intervention, visual inspection of symptom trajectories was used to identify post hoc a data-driven breakpoint at which error bars became overlapping, visible at greater than or equal to 120 days postinfusion (Figure 2). To minimize multiple comparisons, pairwise planned contrasts were then used to probe group means during these data-driven early (≤90 days) and late (≥120 days) timeframes.

## Results

Among 154 randomized participants, 97 (63.0%) were assigned female sex at birth, and 57 (37.0%) were assigned male sex at birth; 11 (7.1%) self-identified as Hispanic or Latino, 9 (5.8%) as Asian, 7 (4.6%) as Black, 116 (75.3%) as non-Hispanic White, and 11 (7.1%) as more than 1 race; the mean (SD) age was 34.3 (10.5) years; and they had a mean (SD) of 2.64 (1.93) prior antidepressant treatment failures.

A significant time point × group interaction was observed in the full model (*P *for interaction < .001). As illustrated in Figure 2, in the early timeframe (defined post hoc as <120 days), there was a significant effect of group for ketamine plus ASAT vs saline plus ASAT (β  = −0.37 [95% CI, −0.71 to −0.04]; *P* = .03), while there was no corresponding significant effect when ketamine was given without ASAT (ketamine plus sham) compared with saline plus ASAT (β = −0.20 [95% CI, −0.54 to 0.14]; *P* = .25). By contrast, in the late timeframe, there were no longer any significant differences between groups (ketamine plus ASAT vs saline plus ASAT: β = 0.18 [95% CI, −0.15 to 0.51]; *P* = .29; ketamine plus sham vs saline plus ASAT: β = 0.11 [95% CI, −0.23 to 0.45]; *P* = .52).

## Discussion

Our study found that the rapid effects of ketamine can be made more enduring with simple, portable, digital techniques that would be relatively easy to provide to patients in a wide range of settings. One 40-minute drug administration, followed by 4 days (30-40 min/d) of digital exercises, created a statistical effect on depression that endured for 3 months according to the new follow-up phase data presented here—even in the context of naturalistic treatment changes across all groups. However, this benefit was not sustained in months 4 to 12.

Study limitations include lack of a no-treatment (saline plus sham) group, precluding conclusions regarding the effect of ASAT as a standalone intervention given that all participants received at least 1 active intervention component, and low base rates of clinically impactful outcomes (eg, suicidal events). Future research is needed to make the initial effect size both larger (given persistent mild-to-moderate average depression levels observed in all study groups) and more durable, potentially through repeated drug administrations, booster sessions of the digital therapy (including at-home administration), and/or additional conditioning modules to target other implicit cognitive patterns. Similar techniques could be tested for their potential to produce rapid, efficient, non–resource-intensive, and relatively enduring relief for a wide variety of common psychological conditions, including suicidality, anxiety, and more.

## Notes

Supplement 1.
Trial Protocol

Supplement 2. **eMethods.** Supplemental Methods and Materials
eReferences

Supplement 3.
Data Sharing Statement
