# Supplementary material for: One-Year Outcomes Following Intravenous Ketamine Plus Digital Training Among Patients with Treatment-Resistant Depression: A Secondary Analysis of a Randomized Clinical Trial
Source: JAMA Netw Open. 2023 May 8;6(5):e2312434. doi: 10.1001/jamanetworkopen.2023.12434 (PMC10167566; doi:10.1001/jamanetworkopen.2023.12434)
Supplement: Supplement 1. — Trial Protocol [file jamanetwopen-e2312434-s001.pdf]

Study Protocol

Official Title: Intravenous Ketamine Plus Neurocognitive Training for Depression

ClinicalTrials.gov ID (NCT number): NCT03237286

Initial IRB approval: 08/14/2017

Protocol Date: 08/07/2020; Amended/modified version (study exclusion #19 added)

Scientific Background

Ketamine is a glutamatergic agent used routinely for induction and maintenance of anesthesia. In randomized controlled trials, subanesthetic (typically, 0.5mg/kg) intravenous ketamine exhibits well-replicated, rapid, potent (i.e., metaanalytic Cohen's d=1.4, a large effect) antidepressant effects, even in difficult-to-treat conditions such as treatment-resistant depression and bipolar depression. Antidepressant effects begin app. 2 h post-infusion (after acute dissociative and euphoric side effects subside) and continue far beyond the drug's elimination half-life of 2.5-3h. However, effects also dissipate rapidly, with efficacy over placebo fading by 7 days post-infusion. To date, the only strategy shown in replicated datasets to extend ketamine's rapid effects is to give continued ketamine infusions; while some clinicians now offer this service directly to patients, there are substantial feasibility and safety concerns for long-term use, including potential neurocognitive impact and neurotoxicity, dissociative/psychotomimetic side effects, addiction/abuse liability, and burden on patients (e.g., high out-of-pocket expense in community settings) and the healthcare system (e.g., intravenous administration; legal constraints given US schedule III controlled substance status). Alternative administration routes (e.g., intranasal) are under development, but do not circumvent many of these challenges. Attempts to develop alternative pharmaceuticals that either reproduce ketamine's rapid antidepressant effects or extend them after an initial ketamine infusion (e.g., ketamine followed by oral D-cylcoserine, which showed promise in a case series of 7 patients) are advancing rapidly, but so far no controlled study demonstrates both rapidity and durability in patients. While these avenues hold tremendous potential, psychiatric care is likely to benefit from a diversity of approaches, including those that efficiently mobilize learning mechanisms to generate gains that are both rapid and enduring.

The rapidity and magnitude of ketamine's effects on depressive-like behavior are attributed to its ability to rapidly and profoundly reverse neuroplasticity deficits. According to neuroplasticity models of depression, chronic stress leads to sustained decreases in neuroprotective factors [e.g., brain-derived neurotrophic factor (BDNF) expression and signaling] that damage plasticity, fostering neuronal atrophy and synaptic depression, particularly in the PFC and hippocampus. This results in deficient adaptation to the environment, compromising learning and stress coping, and to downstream gain of activity in some 'salience network' (SN) regions (e.g., amygdala) regulated by the PFC. Conversely, when neuroplasticity is enhanced (e.g., by treatment), synaptic contacts increase, enhancing adaptability by allowing activity-dependent competition to stabilize the neural structures that best represent internal and external conditions.

A rigorous animal literature converges on a fundamental role for increases in neuroplasticity in mediating ketamine's effects on depressive-like behavior. While debate continues about the precise molecular cascades involved, a recent breakthrough linked ketamine's antidepressant effects in mice to a specific ketamine metabolite, hydroxynorketamine (HNK), which induces AMPA receptor activation and was both necessary and sufficient to decrease depressive-like behavior. AMPA activation is linked, in turn, to a range of cascading neuroplasticity increases, each previously implicated in ketamine's antidepressant action (e.g., increased BDNF, overall PFC synaptogenesis and synaptic activation). Such effects have been studied primarily in AMPA-rich areas of hippocampus (e.g., CA1) and medial (m)PFC—areas also showing plasticity deficits in depression. Convergently, using rigorous optogenetic and molecular blockade techniques, synaptic potentiation within a ventral hippocampal (vHipp)-mPFC pathway was both necessary and sufficient for ketamine's antidepressant-like effects. However, glutamate

receptors are ubiquitous throughout the brain, suggesting the potential for far-reaching, rapid functional reorganization, as observed in patients and monkeys 24-hrs post-ketamine.
Given that synaptic plasticity involving glutamatergic receptors is considered the major molecular substrate of learning and memory in the brain, these findings suggest the novel hypothesis—testable with the proposed experimental therapeutics design—that ketamine-induced neuroplasticity opens a clinical 'window of opportunity' for new, protective learning. By introducing a Cognitive Training protocol into the neuroplasticity window, we propose to be strategic regarding the neural structures that will be repeatedly activated during the experience-dependent 'competition' phase.

Challenge: Disorders of negative affect (anxiety, depression) are the most prevalent and costly class of mental health condition, with a collective public disease burden of staggering proportions. While efficacious treatments have been available for decades, remission rates are low, relapse rates are high, and disorder prevalence rates remain notably consistent, with only 12.7% of patients receiving minimally adequate treatment. In recent years, two potential breakthroughs have ignited hopes for turning a corner in the clinical management of these disabling disorders. First, rapid-acting pharmacological agents—most notably intravenous ketamine—have shown promise in dramatically reducing symptoms within the space of 2-24 hours, overcoming the sluggish response to conventional therapies (e.g., 4-6 week delays) which prolongs suffering, creates non-compliance issues, and fails to address urgent clinical needs (e.g., suicidality and other psychiatric crises). Second, intervention development has increasingly sought to take advantage of technology to increase patient access, reduce cost, and minimize aversive consequences through the use of automated, computer-based procedures. The current proposal aims to synergistically combine the benefits of these two approaches.
The rapidity of ketamine's effects offers the promise of a breakthrough in the way that disorders of negative affect are managed, but this promise remains unfulfilled to date. Rapidly dissipating effects, coupled with substantial concerns regarding safety and feasibility of repeated infusions, limit ketamine's clinical impact. The standard paradigm in ketamine research, as in much of psychopharmacology research, has been to focus on molecular mechanisms of action that are linked to depressive-like behaviors in pre-clinical animal studies, or in humans using in vivo molecular assays. While molecular effects are critical for pharmacological treatment development, a narrow focus on one level of analysis hinders a comprehensive understanding of the pathophysiology of affective dysfunction and treatment mechanisms, leaving sizable knowledge gaps in the pathway that leads from ketamine's molecular effects to profound, rapid changes in complex human experiences (thoughts, moods). Consistent with the NIMH RDoC and experimental therapeutics initiatives, we propose to fill in these gaps and integrate knowledge across cognitive and negative valence systems by examining ketamine's rapid effects on neural, cognitive and implicit information processing indices.
While exciting and substantial advancements have recently been made in understanding ketamine's molecular mechanisms of action, translation to safe, effective pharmacology can be slow and uncertain. Furthermore, long-term pharmacological intervention is unlikely to be beneficial, feasible, and appealing to every patient. A large majority of patients (e.g., up to 91% of depressed patients enrolling in drug trials) consistently express preference for behavioral or combined behavioral/pharmacological treatments, in part reflecting the belief that such treatments will introduce new learning that will solve the core problem and instantiate lasting change. Pharmacological regimens are challenging to maintain in the long term due to patient

discontinuation and the rarity of follow-up opportunities in community practice. A more integrative model of ketamine's impact—in particular, anticipated increases in flexible processing across cognition, information processing, and neural networks—reveals a previously unforeseen opportunity to test for synergistic effects when ketamine is coupled with a brief, automated cognitive training intervention designed to consolidate beneficial forms of cognitive processing while neuroplasticity is high. Thus, this project will push the boundaries of how rapid-acting agents could be used clinically, i.e. as short-term cognitive flexibility enhancers. Here, rapid-acting pharmacology becomes merely the first step in a novel treatment algorithm designed to foster relief that is both rapid and durable, simultaneously taking advantage of technology's potential for low-cost, portable, safe, dissemination-ready intervention. Consistent with findings suggesting gold-standard behavioral treatments can prevent depression relapse in the absence of ongoing care, and preliminary evidence that brief cognitive training approaches can likewise have enduring effects over 1-year, we propose that the introduction and facilitation of new learning during a ketamine-induced window-of-opportunity will provide an efficient path to relief that is both rapid and enduring.

Study Objectives

This project seeks to identify the neural and cognitive changes that accompany rapid relief from depressive symptoms following intravenous ketamine. We will then test whether these changes promote the uptake of helpful information delivered by a computer-based training protocol. This work could ultimately lead to the ability to treat depression more efficiently by rapidly priming the brain for helpful forms of learning.

Study Design & Methods

This study is a randomized, double-blind, parallel arm, controlled trial. A target sample size of 150 patients exhibiting the target deficits (negative self-representations and elevated depressive symptoms) will be randomized 2:1 to intravenous ketamine (0.5mg/kg) or saline, followed by either a computer-based cognitive training protocol designed to implicitly instill positive self-representations via Evaluative Conditioning (n=100 drawn from either the ketamine or saline groups) or a sham variant (n=50, from the ketamine group only). Both males and females age 18-60 will be enrolled.

All infusions will be given over 40 minutes and administered in the MUH-CTRC by an experienced team with a linked ACLS-certified code team in a medical hospital setting, with safety/adverse event monitoring sustained for 4-hours post-infusion. Patient safety/decompensation will be monitored at every visit using appropriate exit/referral plans. Patients will be instructed to have nothing by mouth (except for water and coffee) the morning of the infusion and until 2 hours post-infusion.

Randomized, blinded, controlled trials provide the most rigorous method to test intervention effects at any level of analysis. A parallel arm design was selected for primary hypothesis tests due to previously observed cross-over effects in ketamine studies(114) and an anticipated increased risk of unblinding (for both ketamine and CT) if participants can directly compare treatment experiences (e.g., side effects, positive vs. neutral CT stimuli). 2:1 ketamine:saline followed by 2:1 CT:sham randomization was selected to increase probability of benefit to participants(115) and improve recruitment given substantial interest in ketamine from patients and referring clinicians, while maximizing statistical power for key comparisons: ket (n=100) vs. saline (n=50); ket+CT (n=50) vs. ket+sham (n=50) vs. saline+CT (n=50). A 1-month acute follow-up period is feasible and ethical for this initial efficacy study and, if supported, would have a clinically relevant impact, reducing safety and feasibility concerns regarding frequent infusions(28); 12m naturalistic follow-up will further characterize durability and inform frequency for future trials.

Participants will not be withdrawn from existing depression treatments (e.g., medications, therapy). They may continue in existing therapies for the duration of the study, provided they meet all inclusion/exclusion criteria.

Summary of data collection timeline:
The timeline below represents the target day for each set of procedures. At the PI's discretion, the precise day of assessments (e.g., number of days post-infusion) may be adjusted slightly in order to accommodate participant and research facility schedules.

All participants will undergo a medical screening and thorough psychiatric evaluation consisting of a structured diagnostic interview as well as self-report questionnaires to determine eligibility for study participation. Screening assessments for exclusion criteria:
• Initial phone screen
• General demographic and medical history forms
• Medical intake and comprehensive screening assessments performed by clinical research staff at Bellefield Towers or 3501 Forbes Ave (Oxford Building). Findings will be reviewed for eligibility and signed by a board-certified physician co-investigator (Dr. Howland) prior to the infusion. A drug test will also be obtained at intake; urine pregnancy tests may be obtained prior to fMRI scans at the MRRC.

Screening/baseline procedures include pre-infusion psychiatric evaluation, medical screening, and assessment battery (depression/emotional functioning assessments, neuroimaging, cognitive tasks)=1-2 visits over approximately 1-3 weeks

Infusion (ketamine or saline) visit ("Intervention Day 1" in consent form)=one visit, completed within approximately 1 week of baseline assessments

24-hours post-drug assessment battery ("Intervention Day 2" in consent form) (depression/emotional functioning assessments, neuroimaging, cognitive tasks)=one visit completed 24-hours after drug infusion

Cognitive training sessions and brief assessment battery ("Intervention Days 2,3,4,5" in consent form) (depression and suicidality assessments)=eight 15-20min sessions completed 2x/day (sessions separated by >=20 min) on days 1-4 post-infusion

Infusion +5-days assessment battery ("Intervention Day 6" in consent form) (depression/emotional functioning assessments, neuroimaging, cognitive tasks)=one visit completed following completion of all cognitive training sessions

Infusion +7 days remote (questionnaire) assessment (depression assessments)

Infusion +12 days assessment battery (depression/emotional functioning assessments, cognitive tasks)

Infusion +21 days remote (questionnaire/phone) assessment (depression and suicidality assessments)

Infusion +30-days assessment battery (depression/emotional functioning assessments, neuroimaging, cognitive tasks)

Infusion +2, 3, 4, 5, 6 & 12-months remote, naturalistic follow-up assessments (depression/emotional functioning assessments)=questionnaires completed monthly 2-6 months post-infusion and then once more at 12-months follow-up

Total duration of acute study period (after screening is completed)=approximately 4-5 weeks. Includes 1 infusion visit, 5 in-person assessment visits, 2 remote assessments, and 8 brief cognitive training sessions (twice-daily for 4 days post-drug infusion).

Total duration of naturalistic follow-up period=1-year.
Naturalistic follow-up visits will occur monthly in months 2-6 post-infusion and again at 12 months post-infusion. In addition to other assessments collected during the acute study period, a comprehensive, community treatment questionnaire will be administered to assess the type, dose and duration of medications, psychotherapy, other depression interventions and total inpatient/outpatient visits.

Eligibility Criteria:

Inclusion Criteria:

Participants will:
1) be between the ages of 18 and 60 years,

2) have not responded to one or more adequate trials of FDA-approved antidepressants within the current depressive episode, determined by Antidepressant Treatment History Form (ATHF) criteria [score ≥ 1]

3) score ≥ 25 on the Montgomery Asberg Depression Rating Scale (MADRS)(123)

4) score >1SD above the normative mean on the Cognitive Triad Inventory (CTI(127)) "self" subscale(128,129), a well-validated self-report measure of maladaptive self-representations *OR* <1SD below the normative mean on the Rosenberg self-esteem scale, a widely used self-report measure of self-esteem

5) possess a level of understanding sufficient to agree to all tests and examinations required by the protocol and must sign an informed consent document

6) agree to sign a release of information (ROI), identifying another individual [friend, family member, etc.] as a contact person while the patient is enrolled in the study.

Exclusion Criteria:

1) Presence of lifetime bipolar, psychotic, or autism spectrum; current problematic substance use (e.g., substance use disorder); or lifetime recreational ketamine or PCP use

2) Use of a Monoamine Oxidase Inhibitor (MAOI) within the previous 2 weeks
3) Failure to meet standard MRI inclusion criteria: those who have cardiac pacemakers, neural pacemakers, cochlear implants, metal braces, or other non-MRI-compatible metal objects in their body, especially in the eye. Dental fillings do not present a problem. Plastic or removable dental appliances do not require exclusion. History of significant injury or surgery to the brain or spinal cord that would impair interpretation of results.

4) Current pregnancy or breastfeeding, or failure to engage in an effective birth control strategy throughout the duration of the study

5) Acute suicidality or other psychiatric crises requiring treatment escalation. We will use the Columbia-Suicide Severity Rating Scale (C-SSRS; Version 1/14/09) as both an initial exclusion criteria (C-SSRS "Baseline/Screening" Version for past 1-month period) and as grounds for rescue/removal (C-SSRS "Since Last Visit" form). The C-SSRS will be administered using a paper form by an experienced and thoroughly trained clinical assessor on the study team. Subjects with C-SSRS suicide ideation scores scored "yes" on items 4 (active suicidal ideation with some intent to act) and/or 5 (active suicidal ideation with specific plan and intent) will be excluded from the study, and if enrolled, will be exited from the study and referred immediately

to the nearest emergency mental health facility for additional thorough assessment and appropriate treatment referral.

6) Changes made to treatment regimen within 4 weeks of baseline assessment

7) Reading level <6th grade as per patient self-report

8) For study entry, patients must be reasonable medical candidates for ketamine infusion, as determined by a board-certified physician co-investigator during study Screening. Serious, unstable medical illnesses including respiratory [obstructive sleep apnea, or history of difficulty with airway management during previous anesthetics], cardiovascular [including ischemic heart disease and uncontrolled hypertension], and neurologic [including history of severe head injury] will be exclusions.

9) Clinically significant abnormal findings of laboratory parameters [including urine toxicology screen for drugs of abuse], physical examination, or ECG.

10) Uncontrolled or poorly controlled hypertension, as determined by a board-certified physician co-investigator's review of vitals collected during screening and any other relevant medical history/records.

11) Patients with one or more seizures without a clear and resolved etiology.

12) Patients starting hormonal treatment (e.g., estrogen) in the 3 months prior to Screening.

13) Past intolerance or hypersensitivity to ketamine.

14) Patients taking medications with known activity at the NMDA or AMPA glutamate receptor [e.g., riluzole, amantadine, memantine, topiramate, dextromethorphan, D-cycloserine], or the mu-opioid receptor.

15) Patients taking any of the following medications: St John's Wort, theophylline, tramadol, metrizamide

16) Patients who have received ECT in the past 6 months prior to Screening.

17) Patients currently receiving treatment with vagus nerve stimulation (VNS) or repetitive transcranial stimulation (rTMS).

18) Patients taking benzodiazepines or GABA agonists (within 8 hours of infusion)

****Revised/additional exclusion criterion added on IRB's instruction, effective 08/07/2020 through the end of study****
19) Patients with diabetes
Rationale: For the duration of the COVID-19 pandemic, patients with diabetes, who are increased risk of serious complications from COVID-19 exposure, will be excluded.

Statistical Considerations and Statistical Analysis Plan

Sample Size
150 depressed adults will be enrolled in the study and randomized to one of the three treatment arms: ketamine + cognitive training (n=50); ketamine + sham training (n=50); or saline + cognitive training (n=50). Enrollment will continue until a final sample is acquired that includes the full target of n=150 patients who complete, at a minimum, (1) an infusion day, (2) a 24-hour assessment visit, and (3) ≥75% of intended Neurocognitive Training (active or sham NT) visits, and will thus be considered to have received their NT allocation.

Power Analyses
Previous studies comparing intravenous ketamine to inert and psychoactive comparators (e.g., midazolam) report large effects at 24-hours (d=.80 for depression; d>3 for brain activation). The PI's previous mechanistic CT study in depression found large effects (d=.7-1.0) on symptom and performance-based cognitive measures. Clinically, we are interested in observation of moderate-large effects. The proposed design will yield 80% power to detect moderate effects based on comparisons of 100 ketamine and 50 saline patients (d≥.49 and r≥.31 using α=.05). Based on comparisons of 50 patients in each of the ketamine+CT, ketamine+sham, and saline+CT conditions and 80% power, effects of d≥.57 will be detectable (using α=.05). Power for primary mixed effects analyses comparing treatment groups, which increases with additional repeated measures, will be higher than for such post-hoc single-point contrasts.

Analytic Plan
Infusion Phase. Mixed models regression with timepoint (baseline, infusion+24-hours) as a continuous within-subject factor, drug condition (ketamine vs. saline) as a fixed between-subject factor, and subject as a random factor will be applied to test hypothesized time*drug interactions on outcomes. For outcomes where time*drug interactions are observed, direction of effects within each drug condition will be disambiguated using baseline vs. post-infusion contrasts.
Neurocognitive Training Phase. Mixed models regression with timepoint (24-hrs to 30 days) as a continuous within-subject factor, condition (ketamine+NT, ketamine+sham, saline+NT) as a fixed between-subject factor, and subject as a random factor will be applied to test hypothesized treatment and time*treatment interactions on outcomes. Saline+CT will serve as the reference group to test for enduring (main) effects of treatment and for time*treatment interactions.
Exploratory analysis 1 will extend mixed models analysis of depressive symptoms into the 12-month naturalistic follow-up using self-report (primary self-report outcome=QIDS-SR).
Exploratory analysis 2 will utilize a validated data-driven approach to identify integrative/combined moderators of CT effects (DV=MADRS slope), quantified as the optimal weighted combination of moderators (via elastic net w/cross-validation) across all primary + exploratory indices (e.g., fMRI, HNK/BDNF levels, etc.) assessed after ketamine (n=100), defining the data-driven, integrative brain state that optimizes benefit from CT.
Sensitivity analyses will be applied to all hypotheses to explore the impact of main and moderating effects of key biological variables: sex (stratified at randomization to ensure ~60%

female within-cell), age; and baseline clinical variables: treatment-resistance (stratified), concurrent medication.

Exploratory analyses will probe for generalization to other neurocognitive indices and self-report measures [e.g., depressive schemas (CTI); PROMIS measures; QIDS-SR; WHO Disability Assessment Scale].
