# Supplementary material for: One-Year Outcomes Following Intravenous Ketamine Plus Digital Training Among Patients with Treatment-Resistant Depression: A Secondary Analysis of a Randomized Clinical Trial
Source: JAMA Netw Open. 2023 May 8;6(5):e2312434. doi: 10.1001/jamanetworkopen.2023.12434 (PMC10167566; doi:10.1001/jamanetworkopen.2023.12434)
Supplement: Supplement 2. — eMethods. Supplemental Methods and Materials eReferences [file jamanetwopen-e2312434-s002.pdf]

Supplemental Online Content

Price RB, Wallace ML, Mathew SJ, Howland RH. One-year outcomes following intravenous ketamine plus digital training among patients with treatment-resistant depression: a secondary analysis of a randomized clinical trial. *JAMA Netw Open.* 2023;6(5):e2312434. doi:10.1001/jamanetworkopen.2023.12434

eMethods. Supplemental Methods and Materials

eReferences

This supplemental material has been provided by the authors to give readers additional information about their work.

© 2023 Price RB et al. *JAMA Network Open.*

eMethods. Supplemental Methods and Materials

Ethical Oversight

The study was performed at the University of Pittsburgh and approved by the Internal Review Board of the University of Pittsburgh STUDY19040414, “Testing a Synergistic, Neuroplasticity-Based Intervention for Depressive Neurocognition”. All participants provided written informed consent prior to any study procedure. The study was further overseen by an external Data Safety & Monitoring Board (DSMB) and was monitored by the Clinical Research Education, Support, and Training (CREST) Program through the funding sponsor (NIMH).

Trial Conduct and Reporting Guidelines

This report follows the CONSORT reporting guidelines for randomized studies. All methods, including the *a priori* designated 12-month naturalistic follow-up period, were pre-defined in the trial protocol. The trial was pre-registered under clinicaltrials.gov NCT03237286.

Participants

Participants were recruited and enrolled from 12/01/2017 to 09/30/2021, with the final follow-up assessment completed by 10/18/2022. Participants randomized to all three study arms had moderate levels of depression at pre-infusion baseline (taken on the infusion morning, prior to any intervention procedure), mean MADRS score=32.78 (SD=5.3), which did not differ as a function of treatment arm.

Inclusion criteria specified that participants: 1) be between the ages of 18 and 60 years; 2) have not responded to one or more adequate trials of FDA-approved antidepressants within the current depressive episode, determined by the Massachusetts General Hospital Antidepressant Treatment Response Questionnaire (ATRQ); 3) score ≥ 25 on the Montgomery Asberg Depression Rating Scale (MADRS)(1); 4) score >1SD above the normative mean on the Cognitive Triad Inventory(2) "self" subscale *OR* <1SD below the normative mean on the Rosenberg Self-Esteem Scale(3), which was used to ensure a mechanistic fit between the

© 2023 Price RB et al. *JAMA Network Open.*

cognitive target of the ASAT intervention (enhancing self-worth) and the depressed patient subpopulation endorsing low self-esteem as a prominent symptom of depression [NOTE: in our screening procedures, <4% of patients meeting all other inclusions/exclusions failed to meet this criterion and were ruled out due to insufficiently low self-esteem (n=7 out of >200 patients screened)—suggesting broad generalizability and applicability of our intervention within the pool of treatment-seeking patients with moderate-to-severe, treatment-resistant depression]; 5) possess a level of understanding sufficient to agree to all tests and examinations required by the protocol and must sign an informed consent document; and 6) agree to sign a release of information (ROI), identifying another individual [friend, family member, etc.] as a contact person while the patient is enrolled in the study. These criteria produced an enrolled sample in which all patients met diagnostic criteria for Major Depressive Disorder (MDD), and all but one had a primary diagnosis of MDD (one patient had a primary diagnosis of Posttraumatic Stress Disorder and a secondary diagnosis of MDD). The most prevalent current comorbid diagnosis in the sample was Generalized Anxiety Disorder (n=52; 33.8%) followed by Social Anxiety Disorder (n=48; 31.2%). DSM-5 diagnoses were established by experienced master's-level (or higher) clinicians using the MINI International Neuropsychiatric Interview(4).

Clinical trial exclusion criteria included the following:

1. Presence of lifetime bipolar, psychotic, or autism spectrum; current problematic substance use (e.g., substance use disorder); or lifetime recreational ketamine or PCP use
2. Use of a Monoamine Oxidase Inhibitor (MAOI) within the previous 2 weeks
3. Failure to meet standard MRI inclusion criteria: those who have cardiac pacemakers, neural pacemakers, cochlear implants, metal braces, or other non-MRI-compatible metal objects in their body, especially in the eye. Dental fillings do not present a problem. Plastic or removable dental appliances do not require exclusion. History of significant injury or surgery to the brain or spinal cord that would impair interpretation of results.

© 2023 Price RB et al. *JAMA Network Open.*

4. Current pregnancy or breastfeeding, or failure to engage in an effective birth control strategy throughout the duration of the study
5. Acute suicidality or other psychiatric crises requiring treatment escalation.
6. Changes made to treatment regimen within 4 weeks of baseline assessment
7. Reading level <6th grade
8. For study entry, patients must be reasonable medical candidates for ketamine infusion, as determined by a board-certified physician co-investigator during study screening. Serious, unstable medical illnesses including respiratory [obstructive sleep apnea, or history of difficulty with airway management during previous anesthetics], cardiovascular [including ischemic heart disease and uncontrolled hypertension], and neurologic [including history of severe head injury] will be exclusions.
9. Clinically significant abnormal findings of laboratory parameters [including urine toxicology screen for drugs of abuse], physical examination, or ECG.
10. Uncontrolled or poorly controlled hypertension, as determined by a board-certified physician co-investigator's review of vitals collected during screening and any other relevant medical history/records.
11. Patients with one or more seizures without a clear and resolved etiology.
12. Patients starting hormonal treatment (e.g., estrogen) in the 3 months prior to Screening. Birth control is not an exclusion.
13. Past intolerance or hypersensitivity to ketamine.
14. Patients taking medications with known activity at the NMDA or AMPA glutamate receptor [e.g., riluzole, amantadine, memantine, topiramate, dextromethorphan, D-cycloserine], or the muopioid receptor.
15. Patients taking any of the following medications: St John's Wort, theophylline, tramadol, metrizamide
16. Patients who have received ECT in the past 6 months prior to Screening.

© 2023 Price RB et al. *JAMA Network Open.*

17. Patients currently receiving treatment with vagus nerve stimulation (VNS) or repetitive transcranial stimulation (rTMS).

Revised/additional exclusion criterion added on IRB's instruction (due to COVID-19 harm mitigation efforts), effective 08/07/2020 through the end of study

18. Patients with diabetes

Descriptive Measurements of Race, Ethnicity, Sex, and Gender

In order to accurately characterize the racial and ethnic distribution of the study sample, participants were asked to self-identify (1) their race and (2) their ethnicity by selecting one or more descriptors from a pre-determined list of options reflective of the US Census and National Institutes of Health reporting categories. Participants were also asked to self-identify (1) their biological sex assigned at birth (reported in the Results section of the Research Letter) and also (2) their gender (reported previously(5)).

Additional Methods and Measures During Follow-up Data Collection

Diligent efforts were made at every timepoint to encourage compliance with follow-up surveys irrespective of ongoing circumstances such as clinical status (e.g., worsening depression). This was done in an effort to ensure the follow-up data would be protected against non-random dropout. These measures included: automated survey reminder invitations emailed repeatedly to each participant until completion; follow-up research staff contacts in the event surveys were not completed promptly; in the event contact could not be re-established, use of patient-provided contact information for an identified locator person (acquired as a condition of study enrollment) who could be contacted to provide information on the patient's whereabouts, status, and/or updated contact information.

© 2023 Price RB et al. *JAMA Network Open.*

Participants were asked to complete a self-report questionnaire at each follow-up assessment that inquired regarding the following events: 1) any hospitalizations, suicide attempts, and/or psychiatric emergency room visits since the last assessment point; and 2) new medication changes or new psychotherapies or other somatic therapies started since the last assessment point. Base rates for item #1 were quite low in the current sample (2.2% of patients reported 1 or more such event at 1 or more follow-up assessment), precluding meaningful analysis as a function of treatment arm. As expected given the naturalistic design, medication and/or psychotherapy changes were much more commonly reported (81.3% of sample reported such changes at one or more follow-up assessment), but their rate and frequency of occurrence was evenly distributed across the three treatment arms (p's≥.17).

Analytic Methods Details

Analyses were performed using R version 3.6 using the *lme4* and *emmeans* libraries. The *a priori* statistical significance level for hypothesis tests was two-tailed p<.05.

The hierarchical linear modeling analyses used in all reported analyses are appropriate for data that are either (1) missing completely at random or (2) when missing data is associated with other observed factors (e.g., severity, treatment). As these factors are included in the reported intent-to-treat regression models, which automatically account for missing data points by estimating trajectories over time separately for each participant, missingness related to such observed factors is "*ignorable*, in the sense that inferences can proceed by analyzing the observed data only, without explicitly addressing a (parametric) form of the missing data mechanism."(6) Thus, neither data that are either missing completely at random, nor missingness related to these observed datapoints, poses a risk to the interpretability or validity of models.

© 2023 Price RB et al. *JAMA Network Open.*

eReferences

1. Montgomery SA, Asberg M: A new depression scale designed to be sensitive to change. Br J Psychiatry 1979;134:382-9
2. Beckham EE, Leber WR, Watkins JT, Boyer JL, Cook JB: Development of an instrument to measure Beck's cognitive triad: the Cognitive Triad Inventory. J Consult Clin Psychol 1986;54(4):566-7
3. Schmitt DP, Allik J: Simultaneous administration of the Rosenberg Self-Esteem Scale in 53 nations: exploring the universal and culture-specific features of global self-esteem. J Pers Soc Psychol 2005;89(4):623-42
4. Sheehan DV, Lecrubier Y, Sheehan KH, Amorim P, Janavs J, Weiller E, Hergueta T, Baker R, Dunbar GC: The Mini-International Neuropsychiatric Interview (M.I.N.I.): the development and validation of a structured diagnostic psychiatric interview for DSM-IV and ICD-10. J Clin Psychiatry 1998;59 Suppl 20:22-33;quiz 4-57
5. Price RB, Spotts C, Panny B, Griffo A, Degutis M, Cruz N, Bell E, Do-Nguyen K, Wallace ML, Mathew SJ, Howland RH: A Novel, Brief, Fully Automated Intervention to Extend the Antidepressant Effect of a Single Ketamine Infusion: A Randomized Clinical Trial. Am J Psychiatry 2022:appiajp20220216
6. Ibrahim JG, Molenberghs G: Missing data methods in longitudinal studies: a review. Test 2009;18(1):1-43

© 2023 Price RB et al. *JAMA Network Open.*
