# Supplementary material for: One-Year Outcomes Following Intravenous Ketamine Plus Digital Training Among Patients with Treatment-Resistant Depression: A Secondary Analysis of a Randomized Clinical Trial
Source: JAMA Netw Open. 2023 May 8;6(5):e2312434. doi: 10.1001/jamanetworkopen.2023.12434 (PMC10167566; doi:10.1001/jamanetworkopen.2023.12434)
Supplement: Supplement 3. — Data Sharing Statement [file jamanetwopen-e2312434-s003.pdf]

Data Sharing Statement

Price. One-Year Outcomes Following Intravenous Ketamine Plus Digital Training Among Patients with Treatment-Resistant Depression. *JAMA Netw Open*. Published May 08, 2023. doi:10.1001/jamanetworkopen.2023.12434

Data
Data available: Yes
Data types: Deidentified participant data, Data dictionary
How to access data: https://nda.nih.gov/
When available: beginning date: 06-30-2023

Supporting Documents
Document types: None

Additional Information
Who can access the data: Researchers whose proposed use of the data has been approved
Types of analyses: For a reasonable scientific purpose that has been specified by the requester
Mechanisms of data availability: with a signed data access agreement
